# What Makes Employees’ Work So Stressful? Effects of Vertical Leadership and Horizontal Management on Employees’ Stress

**DOI:** 10.3389/fpsyg.2020.00340

**Published:** 2020-03-19

**Authors:** Wei Wang, Kiroko Sakata, Asuka Komiya, Yongxin Li

**Affiliations:** ^1^Institute of Psychology and Behavior, Henan University, Kaifeng, China; ^2^Social Psychology Laboratory, Graduate School of Integrated Arts and Sciences, Hiroshima University, Hiroshima, Japan

**Keywords:** ethical leadership, mutual support, mutual monitoring, work stress, Japanese sample

## Abstract

Work stress is a significant problem all over the world. In the present study, from the perspective of the combination of vertical and horizontal management, we investigated the relationships of managerial ethical leadership, mutual monitoring, and mutual support among employees’ work stress levels. A total of 307 white collar employees in Japan were asked to complete an online questionnaire on three separate occasions. The results showed that both ethical leadership and mutual support were negatively related to stress. In addition, mutual support mediated the relationship between ethical leadership and work stress. Further, mutual monitoring moderated the relationship between ethical leadership and work stress: when mutual monitoring was high, stress did not decline with more ethical leadership. These results may suggest that ethical leadership can reduce work stress both directly and through mutual support, indirectly. Additionally, the direct effect may be constrained under high monitoring situations. Practical implications and needed future research are also discussed.

## Introduction

Employees’ work stress is a significant problem for organizations worldwide; for instance, United States organizations spend approximately 300 billion dollars per year addressing the consequences of stress (American Institute of Stress, 2012). In Europe, the cost of work stress has reached 20 billion euros per year (Levi and Levi, 2000). In Japan, in particular, 60.9% of employees have reported feeling strong anxiety or stress in their work (Japanese Ministry of Health, Labour, and Welfare, 2012); the word *karoshi*, which means “overwork death,” comes from Japanese (Kanai, 2009). To tackle the work stress problem, the Japanese government has mandated that organizations with over 50 employees conduct stress checks annually at least once (Ministry of Health, Labour, and Welfare, 2015). Nevertheless, at present, such efforts are failing to decrease Japanese workers’ stress (Ministry of Health, Labour, and Welfare, 2015).

What makes employees’ work so stressful and what factors can buffer employees’ work stress? To address this issue, considerable research has focused on the effects of leadership on followers’ work stress. For instance, Skakon et al. (2010) have reviewed the relationship between leadership and followers’ work stress; in general, leaders’ work stress is positively related to followers’ work stress, while leaders’ behavior (e.g., leader’s supportive behavior, consideration behavior) and leadership style (e.g., transformational leadership) are negatively related to followers’ work stress. Whereas most research has stressed the effects on leadership (vertical management), it is still unknown how followers’ management (horizontal management; Ohno, 2005) functions elevate or reduce stress in the workplace, although it is assumed to strongly encumber workers’ lives (Ohno, 2005). To our knowledge, there are no studies examining how vertical leadership and horizontal management interactively influence employees’ stress. Thus, from the prospective of the combination of vertical and horizontal management, the present study aims to explore the relationships between managerial ethical leadership, mutual monitoring, mutual support among followers, and their work stress.

**FIGURE 1:**
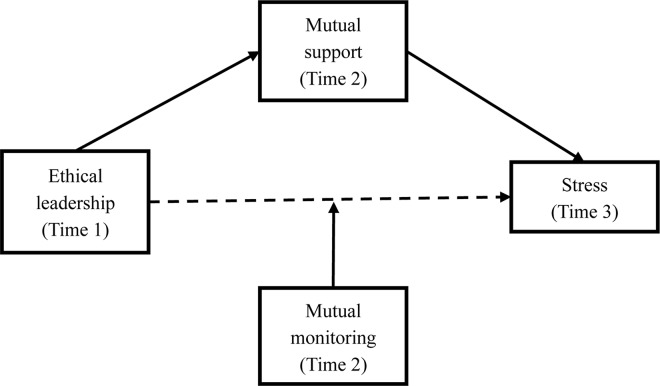
Framework of the present study.

The present study’s purposes are to (a) provide further evidence for the effect of ethical leadership on work stress, by examining the effects of ethical leadership on a series of general stress reactions, including anger, fatigue, anxiety, depression, and physical complaint(s); (b) discuss how ethical leadership can decrease work stress by examining the mediating effect of mutual support between ethical leadership and stress reactions; and (c) discuss conditions that may constrain the effects of ethical leadership, by examining interaction effects of ethical leadership and mutual monitoring on followers’ stress reactions. To examine these potential relations, a time-lagged design was applied in which ethical leadership was measured at time 1, followers’ mutual support and mutual monitoring at time 2, and stress at time 3. The framework of the present study is shown in Figure 1.

### Ethical Leadership and Followers’ Work Stress

From the social learning perspective, ethical leadership is defined as “the demonstration of normatively appropriate conduct through personal actions and interpersonal relationships, and the promotion of such conduct to followers through two-way communication, reinforcement, and decision-making” (Brown et al., 2005). In the definition of Brown et al. (2005), people who are perceived to be ethical leaders model conduct that followers consider to be normatively appropriate (e.g., honesty, trustworthiness, fairness, and care), in turn making the leader a legitimate and credible role model. Ethical leadership comprises two components: the moral person and the moral manager (Brown and Treviño, 2006). On the one hand, ethical leaders themselves exhibit ethical actions and are perceived to be honest, trustworthy, fair, and caring; on the other hand, ethical leaders talk about ethical rules explicitly and make them salient, set ethical standards, and reward ethical conduct—fostering and upholding ethics within their organizations.

Ethical leaders are those who show personal concern for followers and set high ethical standard in organizations (Brown et al., 2005). Thus, ethical leadership is likely to play an important role in organizations that try to thoroughly implement stress prevention guidelines. According to conservation of resources (COR) theory, individuals seek to protect and promote their resources; perception of resource loss, threat to resources, and/or inability to gain new resources can result in stress responses (Hobfoll, 1989). Resources are anything that people personally value: objects, conditions, personal characteristics, energy, etc. (Halbesleben et al., 2009). Ethical leaders show personal concern, respect, and support for followers (Kanungo and Conger, 1993; Treviño et al., 2003), share their power, and provide followers with voice (Yukl, 2006). These aspects of ethical leadership provide important resources to followers, helping them deal with strain in the workplace.

The effectiveness of ethical leadership has gained the attention of researchers and practitioners. Prior work has shown that employee perception of ethical leadership is negatively related to workload, perception of poor conditions, bullying in organizations (Stouten et al., 2010), and unethical acts (Mayer et al., 2012). In addition, ethical leadership is positively related to task significance and job autonomy (Piccolo et al., 2010), task and contextual performance (Brown et al., 2005; Kalshoven and Boon, 2012), satisfaction (Brown et al., 2005), and organizational commitment (Den Hartog and De Hoogh, 2009). Furthermore, based on COR theory, Kalshoven and Boon (2012) and Zheng et al. (2015) have found that by providing resources to followers, ethical leadership is positively related to followers’ well-being and negatively related to followers’ exhaustion. In the present study, depending on the definition of ethical leadership, and consistent with prior studies (e.g., Brown et al., 2005; Stouten et al., 2010), we used the followers’ perception of ethical leadership to measure the ethical leadership. In addition, we used a specific series of stress reactions, including anger, anxiety, fatigue, depression, and physical symptoms as stress indicators, which are well established in Japan (Kawahito et al., 2012). Overall, consistent with prior studies, we predict that:

**Hypothesis 1:** Ethical leadership is negatively related to employees’ work stress, including anger, anxiety, fatigue, depression, and physical symptoms.

### Mediation Effects of Mutual Support

Followers’ work stress is not only affected by vertical management; horizontal management among coworkers may also play an important role. Ohno (2005) suggested that horizontal management consists of two elements: mutual support and mutual monitoring of coworkers. Mutual support was defined as workers supporting one another to achieve occupational goals and supporting one another in various aspects of their private lives; mutual monitoring was defined as workers monitoring one another’s performance, private circumstances, and other aspects of life, and knowing that they are subject to others’ monitoring (Ohno, 2005).

In line with COR theory (Hobfoll, 1989), support from coworkers can provide resources to followers, which can improve their total amount of resources. When employees perceive a loss of resources, threat to resources, or inability to gain new resources, others’ support can compensate for this loss. Therefore, support is expected to be negatively related to stress reactions. In addition, much work has shown that mutual support is positively related to job satisfaction, in-role job performance, organizational citizenship behavior, organizational commitment, and job involvement, and negatively related to burnout, fatigue, job tension, somatic symptoms, and turnover intention (e.g., Cohen and Wills, 1985; Rhoades and Eisenberger, 2002; Viswesvaran et al., 1999). In line with prior studies (e.g., Cohen and McKay, 1984; Jayaratne et al., 1988), we predict that:

**Hypothesis 2***:* Mutual support is negatively related to work stress, including anger, anxiety, fatigue, depression, and physical symptoms.

Based on Hypotheses 1 and 2, both ethical leadership and mutual support may negatively affect work stress. According to social learning theory (Bandura, 1976), leaders are important role models for followers to observe and learn from. Ethical leaders are attractive, credible, and legitimate role models who engage in normatively appropriate behavior and make the ethics message salient (Brown et al., 2005). Followers will then view ethical leaders as role models and learn from them; therefore, the personal concern component of ethical leadership will spread to followers. Followers may then show more concern about their coworkers, fostering mutual support across whole organizations. Therefore, ethical leadership may positively relate to mutual support. Taking Hypotheses 1 and 2 together, we predict that:

**Hypothesis 3**: The relationship of ethical leadership and work stress is mediated by followers’ mutual support.

### Moderating Effects of Mutual Monitoring

Following the definition of mutual monitoring, employees understand their coworkers’ jobs and see their performance in real time (Barron and Gjerde, 1997). Therefore, employees are sensitive to the work status, quality, performance, and errors of their coworkers. On one hand, people in organizations are often reluctant to disclose their errors (Michael, 1976); therefore, they have to pay attention to evaluations of them by others, and this may result in increased tension. On the other hand, mutual monitoring could coerce employees to act according to the “rule” in organizations. The Japan Business Federation (2017) showed that organizations where “overwork is common sense” or “overwork is virtue” fostered longer working hours. If overwork were routinely accepted in high-monitoring organizations, we would expect overwork to become even more accepted, increase, and become a norm. Long working hours, especially unpaid overwork, are positively related with work stress (e.g., Artazcoz et al., 2016; Yamashita et al., 2016); in these ways, mutual monitoring could increase employees’ stress both directly and indirectly, through promoting overwork. Thus, we predict that:

**Hypothesis 4***:* Mutual monitoring is positively related to work stress.

To our knowledge, there are few studies examining the interaction of leadership and monitoring on stress. Based on Hypothesis 1, ethical leadership can reduce followers’ work stress. However, effects of leadership vary from situation to situation (Vroom and Jago, 2007); for instance, Loughry and Tosi (2008) attempted to examined followers’ monitoring in interaction with supervisory monitoring, and their results revealed that when leader’s monitoring was low, direct monitoring (conducted by coworkers) had a positive relationship with performance; when leader’s monitoring was high, direct monitoring (conducted by coworkers) showed no relationship with performance. However, as their work was focused on performance, effects of leadership and mutual monitoring on stress remain unclear.

In the present study, we predicted that ethical leadership may not just directly influence employees’ work stress, but could in fact be constrained by situations in terms of mutual monitoring. As a management function, Ohno (2005) indicated that mutual monitoring among employees is to some extent independent from leadership. Ethical leaders clarify responsibilities, expectations, and performance goals, so that followers know what is expected from them. Meanwhile, through knowledge sharing and communication, mutual monitoring may also form employees’ expectations of their coworkers (Salas et al., 2005). In Japan, no one specific person in an organization generally has the authority and responsibility to make decisions; a bottom-up style of decision-making processes instead prevails (Japan Cabinet Office, 2006), represented by “consensus decision-making” and “delegation of decision-making to middle management” (Pudelko and Mendenhall, 2007). These features of the decision-making process in Japan may be one reason for the stronger influence of horizontal than vertical management. When followers are in low monitoring situations, workers need not to pay much attention to their coworkers’ monitoring, and norms developed by ethical leadership are dominant. Therefore, under low monitoring situations, followers could receive resources provided by ethical leadership and protect their own resources, as ethical leadership is negatively related with stress responses. However, when followers are in high monitoring situations, they both receive monitoring from their coworkers and deliver monitoring to their coworkers, and these two processes together enforce compliance with common rules. Norms shared among coworkers may weaken the effect of ethical leadership. As noted above, overwork is an implicit rule in Japan; when followers face the overwork norm from coworkers and extend their overwork time, they may exhaust resources provided by their ethical leaders or their own resources. Therefore, under high monitoring situations, followers could exhaust resources provided by ethical leadership, and ethical leadership may show no relations with stress responses. Above all, we predict that:

**Hypothesis 5:** The effects of ethical leadership on work stress may be moderated by mutual monitoring.

## Materials and Methods

### Participants and Procedure

This study was conducted among a broad sample of full-time employees in Japan, in a wide range of occupations. Participants were asked to answer a self-reported online questionnaire. The questionnaire used the forced choice approach, ensuring a dataset without missing data. The order of the items and scales was randomized for each participant. In order to test our hypotheses, the surveys were conducted three times; the first time, participants were asked to evaluate their immediate leader; 2 weeks later, they were asked to evaluate mutual support and mutual monitoring in the organization. Then, after 2 weeks, participants were asked to complete the work stress questionnaire.

At time 1, the questionnaires were distributed to 700 employees, and at time 2 and time 3, the 400 employees who returned the time 1 questionnaire were distributed another two questionnaires. In total, 307 employees (144 males and 163 females) completed all three questionnaires. The participants’ average age was 36.0 (*SD* = 7.92). They were mostly working in sales and marketing (18.6%), office/administrative work (14.7%), and technical positions (11.7%). Their average organizational tenure was 8.5 years.

### Measures

#### Ethical Leadership

This was measured with 10 items from the Japanese version of Brown et al. (2005) Ethical Leadership Scale (Watanabe and Sakata, 2014), which is evaluated by followers to assess their direct leaders. An example item is “Listens to what employees have to say.”

#### Mutual Support and Mutual Monitoring

Both mutual support and mutual monitoring were measured by six items from Wang et al. (2016). These items measured followers’ perception of monitoring and support in their organizations. For mutual support, an example item is “We are warm toward members who cannot work enough because of health or family problems.” For mutual monitoring, an example item is “We know we are watched by each other.”

#### Stress Reactions

These were assessed with 26 items from the Brief Job Stress Questionnaire (Kawahito et al., 2012). The participants were asked to assess their stress reactions over the last month. Stress reactions include anger, fatigue, anxiety, depression, and physical symptoms.

#### Others

Demographic items including gender, age, occupation, total number of employees in the company, tenure, and weekly unpaid overwork hours were recorded.

All items were rated on a five-point scale. For stress reactions, this ranged from 1 (*rarely*) to 5 (*frequently*); for other scales, from 1 (*not at all*) to 5 (*very much so*).

### Data Analyses

Data analyses were conducted by SPSS 23.0 with process macro and AMOS 23.0. First, construct validity was examined and the common method variance was examined by confirmatory factor analyses. Then, correlation analyses were conducted to preliminarily examine the hypotheses. Because unpaid overwork is considered to have a strong correlation with stress responses, weekly unpaid overwork hours were controlled in the examination of hypotheses. The mediating effects of ethical leadership and mutual support were examined by hierarchical regression, bootstrap analyses, and structural equation modeling. The moderating effects of ethical leadership and mutual monitoring were examined by hierarchical multiple regression.

## Results

### Construct Validity and Common Method Bias

Although our data were collected at three different times, the measures were evaluated by the same source. Before further testing the hypotheses, the construct validity and common method variance were examined by a series of confirmatory factor analyses. The measurement model—which allowed every item to load on its respective construct—was compared with two nested models. The measurement model consisted of eight factors: ethical leadership, mutual support, mutual monitoring, anger, anxiety, fatigue, depression, and physical symptoms; because mutual monitoring and mutual support were correlated with each other, both theoretically and practically (Salas et al., 2005; Wang et al., 2016), the first nested model combined these two factors. In addition, in the original scoring system of stress responses, items for all stress responses were added together to calculate the total score for stress responses (Kawahito et al., 2012). However, some reports indicated that the effects of antecedent factors may vary on anger, fatigue, anxiety, depression, and physical symptoms (e.g., Murakami et al., 2018). These studies asserted that these stress responses should be discussed separately. Therefore, we compared the factors of the second nested model, which combined these stress response factors. Results of the confirmatory factor analyses are seen in Table 1; as shown, the eight-factor measurement model showed acceptable goodness of fit [χ^2^ (790) = 1635.12, *p* < 0.01, CFI = 0.90, RMSEA = 0.05, SRMR = 0.07] and had a better fit than other models. Combination with the stress reaction factors or the mutual monitoring and mutual support factors showed unacceptable goodness of fit.

**TABLE 1:** Comparison of confirmatory factor analysis.

Models	χ^2^	*df*	Δχ^2^	CFI	RMSEA	SRMR
8-factor model (measurement model)	1635.12	790	−	0.90	0.05	0.07
7-factor model (combining mutual monitoring and mutual support)	1932.55	803	297.43	0.85	0.07	0.29
4-factor model (combining stress reaction factors)	2344.45	815	411.90	0.80	0.08	0.11

Following the suggestion of Podsakoff et al. (2003), the unmeasured latent method construct technique was also applied. On the basis of the original eight-factor structure, a latent method factor was constructed, and all items were allowed to load on it. The latent factor was uncorrelated with other factors. The variance explained by the latent method factor was 4%, which is lower than the 25% median score in published studies (Williams et al., 1989). Furthermore, when constraining the latent method factor’s regression weight to 0, or setting estimation as free, the model fit does not change significantly (Δχ^2^ = 39, n.s.; Richardson et al., 2009). These results provide further evidence that common method variance had little effect on the present study’s overall results.

### Descriptive Statistics and Correlation Analyses

Table 2 presents descriptive statistics, Cronbach’s α, McDonald’s omega, and an intercorrelation matrix. As shown, ethical leadership generally had negative correlations with stress reactions (*r*s = −0.13 to −0.28, *p* < 0.01); these results were consistent with Hypothesis 1. In line with Hypothesis 2, mutual support generally showed negative correlations with stress reactions (for anger, fatigue, anxiety, and depression, *r*s = −0.20 to −0.28, *p* < 0.01; and for physical symptoms, *r* = −0.15, *p* < 0.05). Mutual monitoring was negatively correlated with depression (*r* = −0.15, *p* < 0.05) and slightly negatively correlated with fatigue (*r* = −0.10, *p* < 0.10) and physical symptoms (*r* = −0.11, *p* < 0.10), consistent with Hypothesis 4.

**TABLE 2:** Descriptive statistics and their correlations.

		*M*	*SD*	①	②	③	④	⑤	⑥	⑦	⑧	⑨	⑩	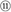
①	Gender	1.53	0.50	–										
②	Age	35.99	7.92	–0.01	–									
③	Unpaid overwork hours	3.13	6.00	−0.13*	−0.10*	–								
④	Ethical leadership	3.19	0.88	0.00	–0.05	–0.03	(0.95)							
							(0.95)							
⑤	Mutual monitoring	2.93	0.59	0.10*	0.04	0.01	0.50**	(0.70)						
								(0.73)						
⑥	Mutual support	3.44	0.72	–0.04	–0.03	0.07	0.26**	0.29**	(0.87)					
									(0.87)					
⑦	Anger	2.26	0.88	0.05	0.03	0.08	−0.25**	−0.21**	–0.05	(0.86)				
										(0.86)				
⑧	Fatigue	2.30	0.89	0.12*	–0.04	0.14**	−0.14**	−0.12*	–0.07	0.61**	(0.88)			
											(0.88)			
⑨	Anxiety	2.05	0.83	–0.03	–0.02	0.07	−0.17**	−0.14**	–0.04	0.62**	0.68**	(0.80)		
												(0.82)		
⑩	Depression	1.96	0.73	0.01	–0.07	0.07	−0.18**	−0.22**	−0.10*	0.67**	0.75**	0.80**	(0.88)	
													(0.89)	
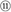	Physical complaint	2.04	0.64	0.20**	0.00	0.05	−0.11**	−0.10*	–0.07	0.48**	0.62**	0.54**	0.62**	(0.71)
														(0.76)

Gender: 1 = male, 2 = female. ** p < 0.01, * p < 0.05. Values in brackets are Cronbach’s α (upper row) and McDonald’s ω.

### Mediating Effect of Mutual Support

In order to further test our hypotheses and better understand the effect of each independent variable, we conducted a series of hierarchical regression analyses for each of ethical leadership, mutual support, and work stress. In step 1, control variables (age, gender, and weekly unpaid overwork hours) were entered into the model; in step 2, mutual support was entered; and in step 3, ethical leadership was entered. When the other demographic variables were entered, the results did not show notable change. As controlling too many variables would decrease the power of the analysis (Becker, 2005), in the present analysis, we used only age and gender as control variables. The results are shown in Table 3.

**TABLE 3:** Effects of ethical leadership and mutual support on stress reactions.

		β					*R*^2^	Δ*R*^2^	*p*
		Gender	Age	UOH	EL	MS			
Anger	Step 1	0.05	0.02	0.09^+^			0.01		0.26
	Step 2	0.05	0.01	0.07	−0.21**		0.06	0.04	0.00
	Step 3	0.07	0.02	0.06	−0.14*	−0.14**	0.07	0.01	0.02
Fatigue	Step 1	0.14*	–0.04	0.18**			0.05		0.00
	Step 2	0.14*	–0.04	0.18**	−0.11**		0.06	0.01	0.03
	Step 3	0.14*	–0.05	0.17**	–0.07	–0.08	0.07	0.00	0.18
Anxiety	Step 1	–0.01	0.01	0.12**			0.02		0.11
	Step 2	–0.01	0.02	0.11*	−0.14**		0.04	0.02	0.07
	Step 3	–0.00	–0.02	0.11*	−0.10^+^	–0.07	0.04	0.00	0.22
Depression	Step 1	0.03	–0.06	0.15**			0.03		0.02
	Step 2	0.03	–0.07	0.14**	−0.16**		0.05	0.03	0.00
	Step 3	0.04	–0.06	0.13*	–0.08	−0.17**	0.07	0.02	0.00
Physical complaints	Step 1	0.22**	0.00	0.12**			0.06		0.00
	Step 2	0.25**	0.05	0.12**	–0.02		0.06	0.00	0.65
	Step 3	0.26**	0.06	0.11*	0.05	−0.14**	0.07	0.01	0.02

The numbers are standardized coefficients. UOH, unpaid overwork hours; EL, ethical leadership; MS, mutual support. ** p < 0.01, * p < 0.05, ^+^p < 0.10.

In step 2, ethical leadership was negatively related with anger (β = −0.21, *p* < 0.01, Δ*R*^2^ = 0.04, *p* < 0.01), fatigue (β = −0.11, *p* < 0.01, Δ*R*^2^ = 0.01, *p* < 0.05, anxiety (β = −0.14, *p* < 0.01, Δ*R*^2^ = 0.02, *p* < 0.10), and depression (β = −0.16, *p* < 0.01, Δ*R*^2^ = 0.03, *p* < 0.01). Therefore, Hypothesis 1 was generally supported.

**FIGURE 2:**
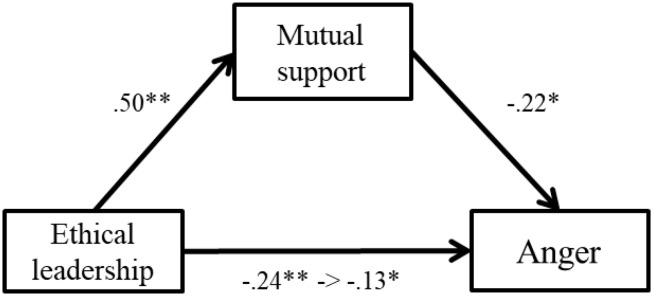
Standardized regression coefficients for the relationship between ethical leadership and anger as partially mediated by mutual support. ^∗∗^*p* < 0.01, ^∗^*p* < 0.05.

**FIGURE 3:**
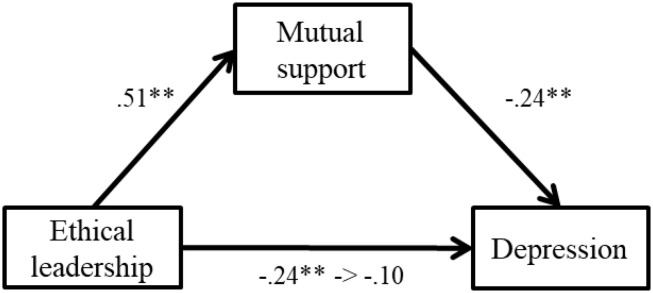
Standardized regression coefficients for the relationship between ethical leadership and depression as completely mediated by mutual support. ^∗∗^*p* < 0.01.

In step 3, controlling for ethical leadership, mutual support showed negative relations with stress reactions and explained additional *R*^2^. Results were as follows: anger (β = −0.14, *p* < 0.01, Δ*R*^2^ = 0.02, *p* < 0.05), depression (β = −0.17, *p* < 0.01, Δ*R*^2^ = 0.02, *p* < 0.01), and physical symptoms (β = −0.14, *p* < 0.01, Δ*R*^2^ = 0.01, *p* < 0.05). However, mutual support showed no relations with fatigue and anxiety (β = −0.07, n.s.; β = −0.07, n.s.). These results suggest that Hypothesis 2 was partially supported. Furthermore, when mutual support was entered into the regression model, the relations between mutual support and stress became weaker than in the prior model. This result suggests that mutual support may mediate the relation between ethical leadership and stress; therefore, we conducted a series of bootstrap analyses (*N* = 2000) for this potential relation. Results are shown in Figures 2, 3.

The relationships between ethical leadership and depression was completely mediated by mutual support. The indirect effect was significant (*B* = −0.08, *SE* = 0.04, *p* < 0.05, 95% CI [−0.18, −0.01]). The relationship between ethical leadership and anger was partially mediated by mutual support. The indirect effect was significant (*B* = −0.13, *SE* = 0.07, *p* < 0.05, 95% CI [−0.20, −0.04]). Therefore, Hypothesis 3 was partially supported.

**FIGURE 4:**
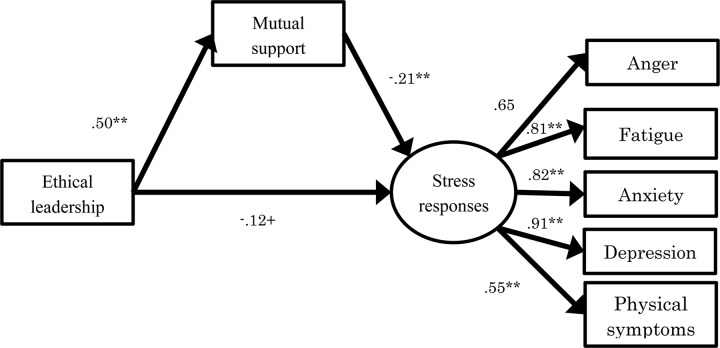
Result of relationship between ethical leadership and stress responses mediated by mutual support [χ^2^ (13) = 34.03, *p* < 0.01. CFI = 0.97, RMSEA = 0.08, SRMR = 0.04]. **p < 0.01, ^+^*p* < 0.10.

To further examine Hypothesis 3, with gender, age, and weekly unpaid overwork hours as control variables, ethical leadership as dependent variable, mutual support as mediating variable, and stress responses took its five dimensions as independent variables, structural equation modeling was conducted. The indirect effect was estimated by bootstrap (*N* = 2000). The model showed acceptable goodness of fit [χ^2^ (13) = 34.03, *p* < 0.01, CFI = 0.97, RMSEA = 0.08, SRMR = 0.04]. The results are shown in Figure 4. As shown, ethical leadership showed a slight negative relation with stress responses and a positive relation with mutual monitoring (β = 0.50, *p* < 0.01). Mutual monitoring showed a negative relation with stress responses (β = −0.21, *p* < 0.01). Mutual monitoring mediated the relationship between ethical leadership and stress responses; the indirect effect was significant (*B* = −0.07, *SE* = 0.02, *p* < 0.01, 95% CI [−0.11, −0.02]). Therefore, Hypothesis 3 was supported.

### Moderating Effect of Mutual Monitoring

In order to test Hypotheses 4 and 5, another hierarchical regression analysis was conducted. In step 1, control variables (age, gender, and weekly unpaid overwork hours) were entered into the model; in step 2, ethical leadership was entered; in step 3, mutual monitoring was entered; and in step 4, the two-way interaction term of ethical leadership and mutual monitoring was entered. The results are shown in Table 4.

**TABLE 4:** Effects of ethical leadership and mutual monitoring on stress reactions.

		β						*R*^2^	Δ*R*^2^	*p*
		Gender	Age	UOH	EL	MM	textbfEL* MM			
Anger	Step 1	0.05	0.02	0.09^+^				0.01		0.27
	Step 2	0.05	0.01	0.07	−0.21**			0.06	0.04	0.00
	Step 3	0.05	0.01	0.07	−0.21*	0.00		0.06	0.00	0.93
	Step 4	0.05	0.01	0.07	−0.21*	0.01	0.08	0.06	0.01	0.12
Fatigue	Step 1	0.14*	–0.04	0.18**				0.05		0.00
	Step 2	0.14*	–0.04	0.18**	−0.11**			0.06	0.01	0.03
	Step 3	0.14*	–0.05	0.18**	−0.09^+^	–0.05		0.07	0.00	0.31
	Step 4	0.13*	–0.04	0.18**	−0.10^+^	–0.05	0.08	0.07	0.01	0.13
Anxiety	Step 1	–0.01	–0.01	0.12*				0.02		0.11
	Step 2	–0.01	–0.02	0.11*	−0.14**			0.04	0.02	0.01
	Step 3	–0.01	–0.02	0.11*	−0.13**	–0.02		0.04	0.00	0.75
	Step 4	–0.02	–0.02	0.11*	−0.14**	–0.01	0.15**	0.06	0.02	0.00
Depression	Step 1	0.03	–0.06	0.15**				0.03		0.02
	Step 2	0.03	–0.07	0.14**	−0.16**			0.05	0.02	0.00
	Step 3	0.02	–0.07	0.15**	−0.14**	–0.08		0.06	0.01	0.13
	Step 4	0.02	–0.07	0.14**	−0.14**	–0.07	0.13*	0.08	0.02	0.01
Physical complaint	Step 1	0.22**	0.00	0.12*				0.06		0.00
	Step 2	0.22**	0.00	0.12*	–0.02			0.06	0.00	0.64
	Step 3	0.21**	0.00	0.12*	–0.07	–0.08		0.06	0.01	0.16
	Step 4	0.21**	0.00	0.12*	–0.01	–0.06	0.16**	0.09	0.03	0.00

The numbers are standardized coefficients. UOH, unpaid overwork hours; EL, ethical leadership; MM, mutual monitoring. ** p < 0.01, * p < 0.05, ^+^p < 0.10.

Mutual monitoring showed no relation with stress reactions; therefore, Hypothesis 4 was not supported. Effects of the interaction between ethical leadership and mutual monitoring on stress reactions were significant for anxiety, depression, and physical symptoms (βs = 0.13–0.16, *p*s < 0.05). Therefore, Hypothesis 5 was partially supported.

**FIGURE 5:**
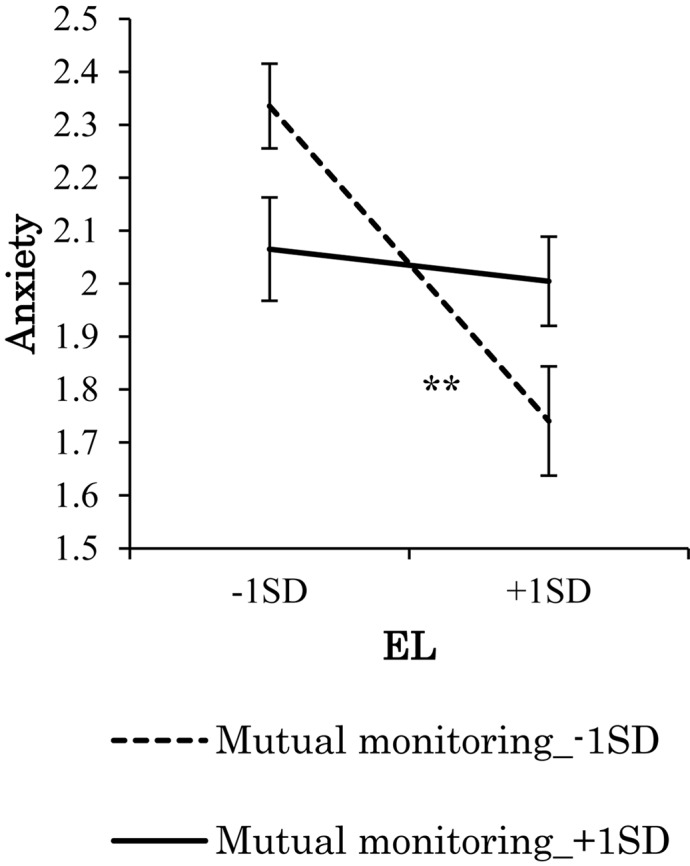
Effect of interaction of EL and mutual monitoring on anxiety. ^∗∗^*p* < 0.01.

**FIGURE 6:**
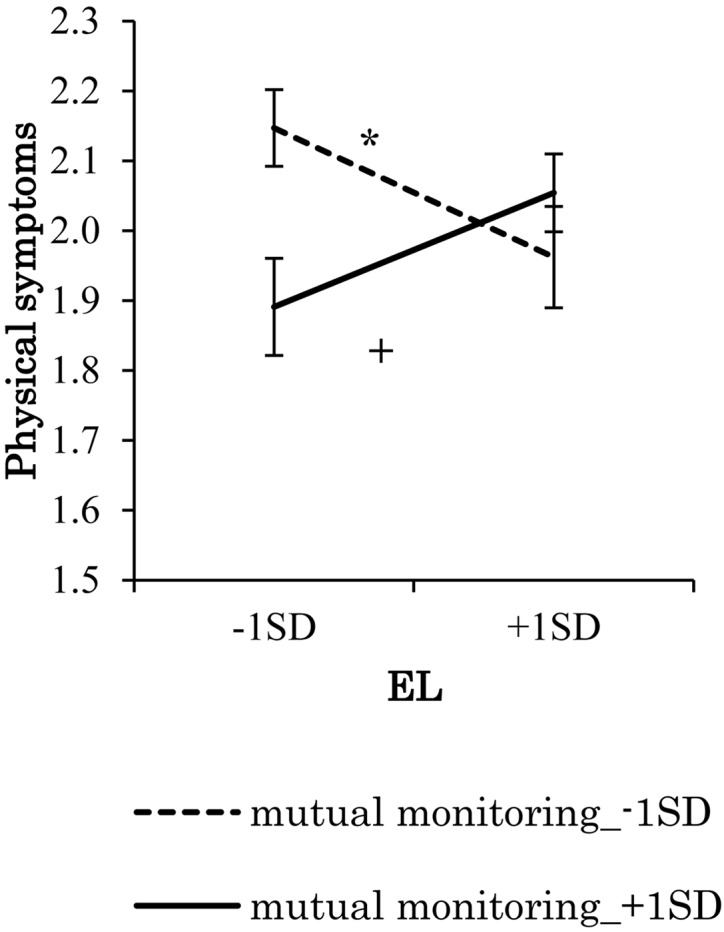
Effect of interaction of EL and mutual monitoring on physical symptoms. **p < 0.01, ^+^*p* < 0.10.

Because the interaction effects of mutual monitoring and ethical leadership on stress reactions were significant, a series of simple slope tests was conducted. The results showed that when mutual monitoring was low, anxiety declined with more ethical leadership, while when mutual monitoring was high, anxiety did not decline with more ethical leadership (Figure 5). Simple slope test results for depression showed the same pattern as anxiety. In addition, simple slope test results of physical symptoms were somewhat different: when mutual monitoring was low, physical symptoms declined with more ethical leadership, however, when mutual monitoring was high, physical symptoms increased slightly with more ethical leadership (Figure 6). Overall, the effects of ethical leadership on stress reactions were constrained by mutual monitoring; therefore, Hypothesis 5 was generally supported.

### Supplementary Analyses

When we determined the construct validity of the model, some model goodness-of-fit indexes were slightly higher than the recommended criterion (i.e., SRMR = 0.07; Wu, 2009). A curvilinear relation could decrease goodness of fit (Kaplan, 1988, 1989), and some studies have suggested that the relationship between ethical leadership and organizational citizenship behavior could be curvilinear (e.g., Stouten et al., 2013). Therefore, we examined the potential relationship between squared ethical leadership and mutual support.

Based on the CFA model, we added the squared ethical leadership items to this model, allowing them to load a new latent factor and this factor to correlate with other latent factors. When the new quadratic factor was added into the model, the model fit improved (χ^2^ = 2424.54, *df* = 1238, CFI = 0.93, RMSEA = 0.05, SRMR = 0.05). Hierarchical regression analysis was conducted to examine the potential curvilinear relation between ethical leadership and mutual support. The result is shown in Table 5.

**TABLE 5:** Curvilinear relation between ethical leadership and mutual support.

	Step 1	Step 2	Step 3
Gender	0.11*	0.10*	0.09*
Age	0.04	0.07	0.07
Ethical leadership		0.51**	0.54**
Ethical leadership^2^			0.10*
*R*^2^	0.01^+^	0.27**	0.28**
ΔR^2^	–	0.25**	0.01*

The numbers are standardized coefficients. ** p < 0.01, * p < 0.05, ^+^p < 0.10.

Ethical leadership was significantly related to mutual support (β = 0.51, *p* < 0.01); the quadratic effect of ethical leadership was also significant (β = 0.10, *p* < 0.05, Δ*R*^2^ = 0.01, *p* < 0.05). Therefore, the curvilinear relation between ethical leadership and mutual support was significant.

To examine whether the curvilinear effect would influence our conclusion of the mediation effects, a series of bootstrap analyses (*N* = 2000) was conducted. Age, gender, weekly unpaid overwork hours, and squared ethical leadership were controlled. The relationship between ethical leadership and depression was completely mediated by mutual support. The indirect effect remained significant (*B* = −0.08, *SE* = 0.03, *p* < 0.05, 95% CI [−0.14, −0.02]). The relationship between ethical leadership and anger was partially mediated by mutual support. The indirect effect remained significant (*B* = −0.08, *SE* = 0.03, *p* < 0.05, 95% CI [−0.15, −0.02]). It can therefore be concluded that the curvilinear effect did not change our results notably.

## Discussion

### General Discussion

Japan is one of the most stressful countries in the world (Ministry of Health, Labour, and Welfare, 2018). The present study took Japanese workers as a sample and examined the effects of both leadership from supervisor and horizontal management from coworkers with a time-lagged design. Specifically, the direct effect of ethical leadership on work stress, the mediating effect of mutual support, and the moderating effect of mutual monitoring were examined. Generally, the hypotheses were supported, and the time-lagged design helped us determine the causal relations between these variables to some extent.

The results were consistent with prior studies in which ethical leadership relates negatively to stress reactions (e.g., Zheng et al., 2015), and these effects were mediated by mutual support. Further, the mediating effects were supported by bootstrap analyses and SEM, which suggested that these results were robust. These results reveal that ethical leadership can reduce the work stress, and provided further evidence supporting the COR theory. However, there are also some novel discoveries in the present study. Contrary to our expectation, the relation between ethical leadership and physical symptoms was not significant. Physical symptoms were chronic stress reactions, which indicate exposure to strain over a long time. Based on social exchange theory, followers working with ethical leaders are more willing to contribute to the organization and have higher work involvement (Den Hartog and Belschak, 2012). Therefore, followers may invest more resources of their own into the organization; this may potentially positively relate to work stress and counteract the negative effects of ethical leadership. Future studies need to test this potential relation.

Contrary to our expectation, mutual monitoring showed no relationship with stress. One possible reason is that mutual monitoring, as an important team function, is not done just to keep tabs on each other but also for better understanding of coworkers’ needs and to provide them with suitable help, encourage coworkers to conform to standards, and decrease errors in organizations (Ohno, 2005; Salas et al., 2005). Furthermore, mutual monitoring motivates and enables workers to engage in behaviors that are beneficial to the organization, that is, to detect opportunities to assist, motivate, or encourage poorly performing coworkers or compensate for their poor performance (LePine and Van Dyne, 2001; Loughry and Tosi, 2008). These components of mutual monitoring may provide resources to their coworkers and was negatively related to stress reactions. Meanwhile, being closely monitored may also connect with stress; Zhou (2003) showed that close monitoring by leaders may cause employees to be preoccupied with task-irrelevant concerns and fears, and it is plausible that being monitored by coworkers may have the same effect. Furthermore, as noted above, mutual monitoring involves setting standards and observing coworkers’ results, and this may lead to a high standard among followers, as these components of mutual monitoring may exhaust their resources. Therefore, taking these findings together, mutual monitoring showed no relation overall with stress reactions.

The results of interactions between ethical leadership and coworkers’ mutual monitoring expand prior ethical leadership research by revealing that monitoring did suppress the effects of ethical leadership on work stress. Both ethical leadership and mutual monitoring had management function aspects. Ethical leadership provided resources to followers, to buffer stress in the workplace. These effects were significant in the low monitoring situation. However, when mutual monitoring was high, the negative relations between ethical leadership and stress disappeared. This result was consistent with the work of Loughry and Tosi (2008), in which followers’ performance increased with higher monitoring from leaders or from followers’ monitoring, however, it did not increase with the combination of high level of leader’s monitoring and high level of followers’ monitoring. As a fundamental team skill, monitoring in organizations means mature cooperation and teamwork to execute some of the leader’s functions. Ethical leaders show concern about followers’ health and make efforts to decrease overwork. However, followers may facilitate another standard: that overwork is normal, and that “one who goes home when everyone is working overtime is not ethical.” In this way, standards emerging from ethical leadership may be misunderstood by followers and result in negative consequences. Further work is needed to examine whether the norms shared by colleagues are consistent with the direction of action recommended by ethical leaders, and their effects on work stress.

Because of the decision-making process in Japanese management style (Japan Cabinet Office, 2006; Pudelko and Mendenhall, 2007), in Japan, horizontal management from coworkers may have stronger influence than vertical management from leaders. The present work supported this inference, showing that the effects of ethical leadership on work stress were mediated by mutual support and constrained by mutual monitoring. Further evidence is needed by collecting data from Japanese employees to examine whether horizontal management has stronger influence than vertical management.

### Theoretical and Practical Implications

With the combination of vertical and horizontal management perspectives, the present study contributes in both theoretical and practical areas.

Theoretically, first, prior studies either focused on the effects of leadership in the vertical mode or those of mutual support and mutual monitoring in the horizontal level. The present study connects these two approaches for a combined vertical and horizontal management. Second, whether from the vertical or the horizontal perspective, the present work was based on COR theory to examine the effects on stress responses and thus expanded the range of application of the theory. Third, the combination of vertical and horizontal approaches helped us clarify the effects and the influence mechanism(s) of antecedent factors on work stress, which could improve our understanding of these phenomena in the context of stress research.

Regarding practical lessons, first, leaders should act ethically, which includes integrity, trustworthiness, and honesty (Brown et al., 2005; Kalshoven et al., 2011). Furthermore, leaders should set rules, standards, and codes of conduct, which provide guidelines for ethical behavior (Beu and Buckley, 2001); leaders can also raise subordinates’ awareness of such guidelines, so that followers can know what is expected and act ethically, show concern for their coworkers, and foster mutual support in organizations. Followers should learn from their ethical leaders and keep support and monitoring at an appropriate level; as also suggested by Albon and Jewels (2014), effective teams need members to maintain awareness of team functioning by monitoring fellow members’ work and catching mistakes, slips, or lapses. Third, for stress prevention, organizations should gain a comprehensive view of followers’ stress management and prevention. It is important to ensure that employees know that the purpose of monitoring is to provide suitable support among coworkers and improve their performance. It is best to avoid high levels of ethical leadership and monitoring simultaneously in organizations, and to ensure followers know the negative effects of monitoring, and improve their own psychological safety and that of their coworkers, to make monitoring less stressful. Followers should learn from their leaders’ ethics and provide suitable support to their coworkers. Furthermore, followers should restrain themselves to an appropriate level of mutual monitoring to avoid unnecessary tension with their coworkers.

### Limitations and Future Studies

Some limitations of the research should be noted. First, mutual monitoring and mutual support are fundamental teamwork skills and a dynamic process that includes both monitoring (supporting) others and being monitored (supported) by others, however, the present study used only a single response source. Even though we randomized the order of the items and scales in the questionnaire and employed an unmeasured latent method construct technique to try to avoid common method variance, more data collected from team units and hierarchical linear model analyses could help us gain a better understanding of the effects of mutual monitoring. Future studies should use work group data to examine the effects of mutual monitoring at both individual and group levels.

Second, because our data were collected in Japan, the generalizability of these findings has to be considered carefully. One possible issue is that these results were intrinsically tied to current work circumstances in Japan. In addition, while efforts to reduce long working hours are beginning in Japan, many people still share the attitude that long working hours are the best way to preserve high work quality and take the burden off colleagues; this norm then engenders and is preserved by the institution of mutual monitoring. If this interpretation is correct, the present findings may be applied to other countries in which long working hours are seen as a virtue. Another possibility is that our findings reflect a limitation of ethical leadership regardless of working circumstances and culture. Some studies have demonstrated a curvilinear effect of ethical leadership: when leadership is too ethical, followers decrease organizational citizenship behaviors (Stouten et al., 2013). Along distinct but analogous lines, our study showed that under certain conditions, ethical leadership may lead to worse health for employees, indicating the importance of finding ways to make mutual monitoring less stressful for employees under ethical leaders. Future study is necessary to address this issue.

Third, the initial measurement model did not fit the data well. Our supplementary analysis showed that the quadratic factor lowered the model fit, and the relationship between ethical leadership and mutual support may not be only linear, the curvilinear relationship was also significant. Furthermore, Pierce and Aguinis (2013) suggested that the relationship between positive leadership style and outcome variables may not be simply positive or nsegative; there may be a non-linear relationship as well. They have also proposed that researchers should pay more attention to the non-linear relationship. Our supplementary analysis provided further material for the non-linear relationship. Whereas the initial purpose of this study was to examine the relationships of managerial ethical leadership, mutual monitoring, and mutual support among employees with their work stress, we have not discussed in detail about the curvilinear relation. Whether a curvilinear relationship exists between ethical leadership and other consequence variables, how ethical leadership affects the consequence variables, and the mechanism of curvilinear relationship remains to be explored in future study.

These limitations notwithstanding, the present study provides interesting implications for both ethical leadership and work stress literatures. Prior research has mainly studied the positive outcomes of ethical leadership; our study instead focused on the combination of ethical leadership and horizontal management, and found that in general, effects of ethical leadership on work stress were mediated by mutual support and moderated by mutual monitoring.

## Data Availability Statement

The raw data supporting the conclusions of this article will be made available by the corresponding author, without undue reservation, to any qualified researcher.

## Ethics Statement

This study was approved by the Research Ethics Committee of the Graduate School of Integrated Arts and Sciences, Hiroshima University.

## Author Contributions

Under the direction of KS, WW generated the idea and designed the study, was the principal investigators for the study, and was the primary writer of the manuscript. The study was supported by a grant from KS. The writing and the framework of this manuscript was under the direction of YL and AK. All authors were involved in developing, editing, reviewing, and providing feedback for this manuscript and have given approval of the final version to be published.

## Conflict of Interest

The authors declare that the research was conducted in the absence of any commercial or financial relationships that could be construed as a potential conflict of interest.
